# Effect of Ultrasound on Extraction Efficiency and Functional Properties of *Acacia* Seeds Protein Concentrates

**DOI:** 10.1111/1750-3841.70566

**Published:** 2025-09-23

**Authors:** Laxmi Ghimire, Nedumpillil Unnikrishnan Sruthi, Simon Warwick, Ranil Coorey, Rewati Raman Bhattarai

**Affiliations:** ^1^ School of Molecular and Life Sciences, Faculty of Science and Engineering Curtin University Perth Western Australia Australia

**Keywords:** *Acacia* protein, extraction, techno‐functionality, ultrasound | yield

## Abstract

*Acacia* is an Australian‐Aboriginal underutilized legume that contains high protein content (18%–35%). However, the commercial use of the seed's protein is limited due to minimum protein yield and low techno‐functional properties. This research investigated the effect of ultrasound on protein yield and functionality of concentrated proteins from the seeds of two *Acacia* species (*Acacia microbotrya* and *Acacia cyclops*) and compared them to soybeans. Proteins were extracted from *Acacia* species and soybeans employing both ultrasound‐assisted extraction and extraction without ultrasound, followed by isoelectric precipitation. The protein yield, functional characteristics, and protein structure were evaluated and compared. The protein yield in ultrasound‐treated samples increased by 10.92%, 6.3%, and 7.84% in all protein concentrates (*A. cyclops*, *A. microbotrya*, and soybean), with the highest yield conditions being 80 W, 20 kHz, and 20 min. The emulsifying activity index in ultrasound‐treated soybean protein concentrate was 2.85 m^2^/g, which was lower than ultrasound‐treated *A. cyclops* but higher than ultrasound‐treated *A. microbotrya*. The foaming capacity of ultrasound‐treated *Acacia* samples and soybean protein concentrate increased by a factor of 9–21 compared to their untreated samples. Likewise, foaming stability, protein digestibility, and water‐ and oil‐holding capacities of ultrasound‐treated *Acacia* seed proteins were significantly higher (*p* < 0.05) than untreated proteins. This work demonstrated that ultrasound is an effective technique that improves protein yield and techno‐functionality of *Acacia* seeds, which may favor the revalorization of this legume.

## 1 Introduction

The genus *Acacia* could be a potential alternative source of edible plant proteins, as there is a high global demand to create food products to feed the growing population and meet the consumer demands for plant‐based foods (Sethi et al. 2016). Plant‐based proteins alleviate the ethical concerns and the environmental impacts of animal proteins derived from animal sources (Silva et al. 2020). In addition, increased consumer demand for plant‐based foods, approximately 14% of the global population is vegetarian, a trend that has contributed to a recent growth in the market for legume‐based protein alternatives (Silva et al. 2020). Dairy and dairy products have captured the world's protein market. However, issues, such as cow's milk protein allergy and lactose intolerance, have been growing over a few decades, which has moderately decreased milk product consumption (Lucarini 2017). Plant proteins are, therefore, replacing animal proteins in consumer's diets (Goldstein and Reifen 2022). Plant protein sources have a rich nutritional profile, phytonutrient content, and bioactive properties and are finding wider usage as food ingredients (Affrifah et al. 2023). Plant proteins have potential applications in food systems with several functional characteristics, such as foaming capacity (FC), emulsifying properties, water‐holding capacity (WHC) and oil‐holding capacity (OHC), and solubility (Pietrysiak et al. 2018). These specific properties of legume proteins have generated possibilities for value addition on the commercial scale of food product categories, such as gluten‐free, dairy alternatives, and meat substitutes (Zhang et al. 2024). Adiamo et al. (2019) reported that legume proteins are economically viable, and their nutritional and functional qualities are similar to animal proteins. Consequently, the demand for these plant proteins would increase.

Soybean is one of the earliest leguminous sources of plant‐derived proteins used for human consumption (Bello et al. 2023). It has dominated the world legume protein market due to its relatively high protein content (35%–45%) (Wilson 2012), its positive health effects (Barac et al. 2015), and broader applications in producing mainstream food products (Bello et al. 2023). However, soybean contains allergens (glycinin and β‐glycinin) and antinutrients, such as lectins, trypsin inhibitors, saponin, and phytate (Norozi et al. 2022), that decrease the absorption of nutrients inside the body (Norozi et al. 2022; Sun et al. 2013). Considering plant‐based protein alternatives in the food industry, pea‐protein application is rising in the world market as a viable substitute for soybean protein. Pea protein possesses substantially improved digestibility and greater levels of essential amino acids (Zhou et al. 2023). In addition, pea proteins show high gelation (Rodriguez and Beyrer 2023), water binding, and emulsifying characteristics (Cui et al. 2021). Nonetheless, the allergenic components vicilin and convicilin in peas are also reported as common allergens in many countries (Bar‐El Dadon et al. 2014; Taylor et al. 2021).

*Acacia* is an underutilized Australian native plant containing 18%–35% protein and 34%–41% dietary fiber (Adiamo et al. 2023). It belongs to the family Fabaceae and is found in drier, warmer, tropical, and subtropical parts of the world, such as United States, Asia, and Africa (Adiamo et al. 2019). Nine hundred and sixty species of *Acacia* are found in Australia; among them, only 40 species can be used as a food source (Adiamo et al. 2019). Native Australians have used the edible *Acacia* seeds in their diet for over 1000 years (Adiamo et al. 2019). As *Acacia* seeds are a good source of proteins, it is essential to use available technology to optimally extract and manipulate the microstructure of protein to design new food products. Protein is the most dynamic component in legumes, providing several techno‐functional properties and having numerous uses in food formulations (Ee et al. 2011; Sethi et al. 2016). Currently, at the commercial level, the Australian bush food industry is selling roasted *Acacia* seeds as a value‐added product (Agboola et al. 2012). However, roasting has been reported to disrupt the techno‐functional properties of the protein by altering protein conformation (Agboola et al. 2012). For example, roasted bambara groundnut and sesame seed at temperatures above 80°C for 600–3600 s reduced the flour's emulsifying and foaming properties (Agboola et al. 2012). Yusuf et al. (2008) reported that roasting sesame seed at 80°C for an hour substantially reduced emulsifying activity and FC (Yusuf et al. 2008). Ee (2008) stated that thermal processing of *Acacia* at 200°C for 1800 s altered flour composition and physio‐chemical properties. Therefore, an appropriate nonthermal method for protein modification should be investigated.

In contrast to thermal methods, ultrasound offers considerable benefits of being safe, nontoxic, environment‐friendly, and significantly improving the techno‐functional characteristics, such as emulsifying properties, solubility, WHC and OHC, FC, and foaming stability (FS) of legume proteins (Al‐Ali 2020). Ultrasound is the most effective technology for extracting and modifying legume proteins, making them suitable for several food applications (Noh and Lee 2024). The microstructural stability of *Acacia*‐derived proteins over a pH range of 3–9, ionic strength (0–50 mM Ca^2+^), and temperature (25–90°C) make it an influential protein group and could have significant potential in food applications as a functional food ingredient (Adiamo et al. 2023). Food scientists have shown much interest in further assessment of *Acacia* seed quality due to its excellent nutritional value, high amount levels of bioactive compounds, relatively few anti‐nutritional factors (Chong et al. 2019), and good processing ability (Adiamo et al. 2019).

Therefore, this study aimed to investigate the effect of ultrasound on extraction efficiency and functional properties of proteins isolated from two *Acacia* species (*Acacia cyclops*, *Acacia microbotrya*) and soybean. These species were selected on the basis of their potential antimicrobial property and high protein content, respectively (Chong et al. 2019). The protein was extracted by applying six different ultrasound treatment conditions, and the effects on protein yield were compared. The treatment condition with the highest protein yield was used to evaluate the critical functional properties, comparing treated and untreated protein samples under identical conditions for all three legumes.

## 2 Materials and Methods

### 2.1 Materials

*Acacia* seeds (*A. cyclops* and *A. microbotrya*) were kindly provided by Maalinup Aboriginal Gallery, and soybean seeds were purchased from Coles, Western Australia. All the seeds were ground and passed through a 500 µm sieve to produce flour. The flours were packed into air‐tight containers and stored under refrigerated conditions until further analysis. Distilled deionized water (DDW) was used in all the experiments.

### 2.2 Chemicals

Sodium chloride, sodium dodecyl sulfate, hydrochloric acid, sulfuric acid, boric acid, sodium phosphate buffer, sodium hydroxide, hexane, and phosphate buffer were purchased from Able Westchem, Western Australia. Canola oil and soybean oil were purchased from Coles, Western Australia.

### 2.3 Protein Extraction and Yield

Proteins were extracted using the method of Aguilar‐Acosta et al. (2020) with modifications. *Acacia* and soybean flours were dispersed in deionized water in the ratio 1:10 (w/v) by constant stirring for 30 min, and the pH was adjusted to 9 using 0.1 N NaOH. The ultrasound extraction was carried using an ultrasonic probe (DSA 100‐GL2, USA) by subjecting the alkaline dispersions to different conditions to identify the optimal parameters: (1) 60 W, 20 kHz, 20 min; (2) 80 W, 20 kHz, 20 min; (3) 100 W, 20 kHz, 20 min; (4) 60 W, 20 kHz, 15 min; (5) 80 W, 20 kHz, 15 min; and (6) 100 W, 20 kHz, 15 min. A chiller was connected to the sonication vessel to maintain the slurry temperature below 35°C throughout the treatment. The ultrasound‐treated alkaline extracts were centrifuged (Heraeus Multifuge 3R, Thermo Scientific, Australia) at 1000 × *g* for 15 min at 4°C. The supernatant was collected and pH adjusted to 4.5 (isoelectric point) using 1 N HCl. The protein precipitates were collected after centrifugation (1000 × *g* for 15 min). The pellets were washed three times with DDW to remove all soluble components, and pH was adjusted to 7 using 0.1 N NaOH. Finally, the neutralized protein pellets were dried using a freeze‐drier (ALPHA 1–2 LDplus, Martin Christ, Germany) at −20°C for 48 h. Protein yield was calculated as follows (Bhattarai et al. 2022):

$$\begin{array}{ccc} & & \mathrm{Protein}\;\mathrm{yield}\;\left(\%\right)\\ & & \;=\frac{\mathrm{total}\;\mathrm{weight}\;\mathrm{of}\;\mathrm{protein}\;\mathrm{isolate}\;\left(g\;\mathrm{dry}\;\mathrm{basis}\right)}{\mathrm{total}\;\mathrm{weight}\;\mathrm{of}\;\mathrm{protein}\;\mathrm{in}\;\mathrm{raw}\;\mathrm{material}\;\left(g\;\mathrm{dry}\;\mathrm{basis}\right)}\\ & & \;\times \;100 \end{array}$$

### 2.4 Functional Properties Assessment

The ultrasound‐treated and untreated *Acacia* and soybean protein extracts were assessed for different functional properties using the following methods.

#### 2.4.1 Foaming Capacity (FC) and Foaming Stability (FS) Water

FC and FS were determined following the method of Xiong et al. (2016). Ultrasound‐treated and untreated protein isolate dispersions were prepared in DDW (3% w/v), and 50 mL of this dispersion was homogenized (Ultra‐Turrax, Ika T18, USA) at 8000 rpm for 2 min. The volume of the solution before and after homogenization was recorded. FC was expressed as the increase in volume due to whipping (%):

$$\mathrm{Foaming}\;\mathrm{capacity}\;\left(\%\right)=\frac{V_{2}-V_{1}}{V_{1}}\times 100$$

where *V*_1_ is the volume of solution before homogenization, and *V*_2_ is the volume of solution after homogenization.

To determine FS, changes in foam volume in a graduated cylinder at room temperature were recorded after 60 min:

$$\mathrm{Foaming}\;\mathrm{stability}\;\left(\%\right)=\frac{V_{4}-V_{3}}{V_{3}}\times 100$$

where *V*_3_ is the volume of foam at 0 min, and *V*_4_ is the volume of foam at 60 min.

#### 2.4.2 Emulsifying Activity Index (EAI)

The EAI was determined on the basis of the method of Mokni Ghribi et al. (2015). The EAI of ultrasound‐treated and untreated protein samples was evaluated by preparing emulsions. In brief, 50 mL of 0.5% w/w protein isolate dispersions were homogenized with 2 mL of canola oil at 13,500 rpm for 60 s. From this, 100 µL of the emulsion was taken immediately from the lower end of the tube and diluted in 7.5 mL of 0.1% sodium dodecyl sulfate, and EAI was calculated using the following equation:

$$\begin{array}{ccc} & & \mathrm{Emulsifying}\;\mathrm{activity}\;\mathrm{indices}\left(m^{2}/g\right)\\ & & \;=\left(2\times 2.303\times A_{0}\times N\right)/C\times \phi \times \;10,000 \end{array}$$

where *A*_0_ is the immediate absorbance taken after homogenization, *N* is the dilution factor (150*), *C* is the protein's weight per volume (g/mL), and *ɸ* is the oil‐volume fraction of the emulsion.

#### 2.4.3 Water Holding Capacity (WHC) and Oil Holding Capacity (OHC) Water

The method of Mokni Ghribi et al. (2015) was used with slight modification to determine WHC of the protein concentrates. Three grams of freeze‐dried protein concentrate were dispersed in 25 mL of DDW. The contents were stirred for 30 min at intervals of 5 min to disperse the sample in water and centrifuged at 3000 *g* for 25 min. The supernatants were discarded, and the samples were drained for 25 min to remove the excess water at 25°C. The increase in weight represents the WHC of the protein concentrates.

OHC was measured using the method proposed by Mokni Ghribi et al. (2015). The protein extracts (0.5 g) were mixed with 6 mL of canola oil, and the dispersions were stirred for 1 min, held for 30 min, and centrifuged for 25 min at 3000 *g*. The excess oils were removed by inverting the tubes for 25 min to drain the oil before reweighing.

The WHC and OHC were calculated as grams of water/oil bound per 100 g of the protein concentrates.

#### 2.4.4 Solubility

Protein solubility was measured by using the pH range of 2–12. Protein sample of 100 mg was suspended in 20 mL DDW, and the pH was adjusted using 0.1 N NaOH or HCl solutions. The suspensions were agitated using a metabolic shaker for 1 h at ambient temperature and centrifuged at 8000 × *g* for 15 min. The Kjeldahl method was used to determine the protein content of the supernatant. The solubility profiles were obtained by plotting averages from triplicates of protein solubility in percentage against pH (2, 4, 6, 8, 10, and 12).

#### 2.4.5 In Vitro Protein Digestibility (IVPD)

IVPD of ultrasound‐treated and untreated samples was determined using the following method (El Faki et al. 1984). Approximately 50 g of treated and untreated protein concentrates *Acacia* seeds and soybean were incubated at 37°C with 0.75 mg pepsin (Chem‐Supply, Gillman, SA, Australia) in 7.5 mL of 0.1 N HCl for 3 h. The solution was neutralized with 3.75 mL of 0.2 N NaOH. Following this, 2 mg pancreatin in 3.75 mL of pH 8.0 phosphate buffer was added, and the samples were incubated at 37°C. The undigested protein in 5 mL of digesta was precipitated by adding 25 mL of 10% trichloroacetic acid, and then the sample was centrifuged for 30 min at 1000 × *g* at room temperature. Nitrogen in the supernatant was determined using the Kjeldahl method. The IVPD was calculated using the formula:

$$\begin{array}{c} \mathrm{IVPD}\;\left(\%\right)=\frac{100-[\mathrm{total}\;\mathrm{nitrogen}\;\left(g/100\;\mathrm{mL}\right)-\mathrm{nitrogen}\;\mathrm{in}\;\mathrm{the}\;\mathrm{supernatant}\;\left(g/100\;\mathrm{mL}\right)]}{\mathrm{total}\;\mathrm{nitrogen}\;(g/100\;\mathrm{mL})}\times 100 \end{array}$$

### 2.5 Attenuated Total Reflection–Fourier Transform IR Spectroscopy (ATR–FTIR)

The secondary structure of proteins from *Acacia* and soybean protein concentrates was analyzed using FTIR (Thermo Scientific, Nicolet iS50 ABX, Australia). Data were collected with a resolution of 4 cm^−1^ at a wavelength from 400 to 4000 cm^−1^. The recorded spectra were analyzed using OPUS software (V 7.0, Bruker, Germany). The obtained spectra were vector normalized to the amide I band (1700–1600 cm^−1^) for calculating the second derivatives using a 13 smoothing point Savitzky–Golay smoothing function (Hackett et al. 2015). The number and locations of the underlying bands were identified for curve fitting. The peaks were assigned to different protein conformations, and the area under the curves was determined to estimate the relative changes in position and shape of the secondary structure of the examined proteins.

### 2.6 Protein Profiles Using Sodium Dodecyl Sulfate–Polyacrylamide Gel Electrophoresis (SDS–PAGE)

SDS–PAGE of the *Acacia* and soybean protein concentrates under non‐reducing and reducing conditions was performed according to the method by Wong et al. (2013) using NuPAGE Novex 10% Bis–Tris gels (Invitrogen, Carlsbad, CA, USA). In this method, 5 µg of protein concentrates were diluted to make 10 µL of the final solution by adding NuPAGE sample buffer (Invitrogen) and then loaded onto the gel. Electrophoresis was performed with a constant current at 200 V for 1 h with 2‐morpholinoethanesulfonic acid monohydrate (MES) SDS running buffer (Invitrogen) until the electrophoretic front was 1 cm from the bottom of the gel. Fixed proteins were stained using 50 mL of Bio Safe Coomassie stain (Bio‐Rad Laboratories, Hercules, CA, USA) and then destained using 5% methanol and 7.5% acetic acid solution. The molecular weights (MWs) of the peptide bands in the protein samples were estimated by comparing their migration to bands of the marker's MW (prestained SDS–PAGE standards, broad range, Bio‐Rad).

### 2.7 Statistical Analysis

Triplicate independent determinations were carried out for all the analyses. All the obtained results were expressed as mean ± standard error. The statistical analysis was performed using a statistical software program (SPSS version 17), and one‐way ANOVA was used to determine the significance of the results obtained between ultrasound‐treated and untreated protein samples (*p* < 0.05). The plots were generated using OriginPro 2024b (10.1.5.132 Academic) software package.

## 3 Results and Discussion

**FIGURE 1:**
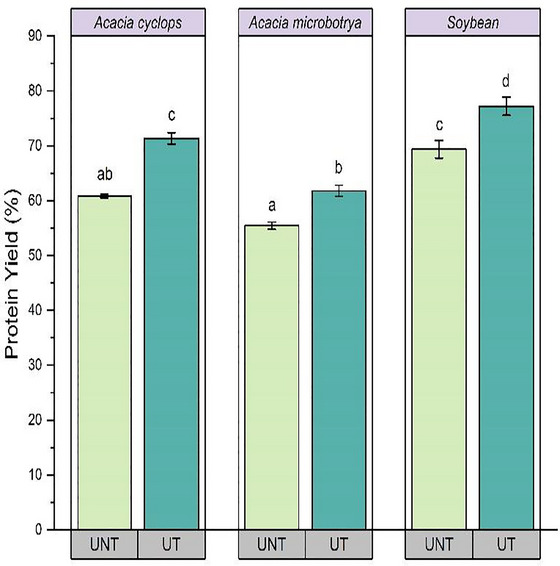
Changes in protein yield of *Acacia cyclops*, *Acacia microbotrya*, and soybean for untreated and ultrasound‐treated conditions. Values in different letters indicate a significantly difference (*p* < 0.05). UNT, untreated; UT, ultrasound‐treated (80 W, 20 kHz, 20 min: highest yield condition).

Table 1 represents both ultrasound‐treated and untreated sample data for the protein yield and functional properties of protein concentrates obtained from *A. cyclops*, *A. microbotrya*, and soybean, and Figure 1 represents the recorded data for both untreated and ultrasound‐treated conditions.

**TABLE 1:** Protein yield and techno‐functional properties of treated and untreated protein concentrates.

Sample	Treatment	Protein yield (%)	Foaming capacity (%)	Foaming stability (%)	IVPD (%)	Emulsifying activity indices (m^2^/g)	Water‐holding capacity (%)	Oil‐holding capacity (%)
*Acacia cyclops*	UNT	60.84 ± 0.31^a^	1.78 ± 0.003^ace^	1.77 ± 0.013^ace^	84.20 ± 0.26^a^	2.39 ± 0.14^a^	72.80 ± 3.22^a^	422.12 ± 7.90^a^
	UT	71.76 ± 1.04 ^b^	37.93 ± 2.73^bdf^	7.88 ± 1.59^bd^	96.72 ± 0.06^b^	5.48 ± 0.27^b^	76.78 ± 1.88^abd^	485.40 ± 17.10^b^
*Acacia microbotrya*	UNT	55.43 ± 0.68^c^	2.31 ± 0.59^cf^	1.92 ± 0.2^ce^	82.25 ± 1.19^ac^	1.75 ± 0.11^c^	73.80 ± 0.55^cde^	216.77 ± 5.78^c^
	UT	61.80 ± 1.04^b^	32.84 ± 2.73^df^	7.51 ± 1.12^d^	93.45 ± 1.74b^d^	2.06 ± 0.12^d^	74.83 ± 2.2^bc^	137.67 ± 5.38^d^
Soybean	UNT	69.36 ± 1.59^e^	3.74 ± 1.14^e^	2.73 ± 0.56^e^	81.13 ± 0.95^ace^	1.48 ± 0.69^e^	72.80 ± 2.14^ef^	177.71 ± 5.38^e^
	UT	77.20 ± 1.65^f^	33.65 ± 4.61^f^	12.31 ± 0.46^f^	90.49 ± 0.59^a^	2.85 ± 0.16^f^	81.34 ± 2.13^f^	285.72 ± 35.34^f^

Abbreviations: IVPD, in vitro protein digestibility; UNT, untreated; UT, ultrasonication treated (80 W, 20 kHz, 20 min: highest yield condition). Values in different letters in each column indicate a significantly difference (*p* < 0.05).

### 3.1 Effect of Ultrasound on Protein Yield

Preliminary tests were conducted to determine the optimal ultrasound condition for high protein yield. The highest protein yields were observed at 80 W power and 20 kHz frequency for 20 min for *A. microbotrya* and soybean, and at 100 W, 20 kHz, 20 min for *A. cyclops*. However, the differences in protein yields at these conditions were not statistically significant (*p* > 0.05). Therefore, 80 W, 20 kHz, and 20 min were selected as the standard ultrasound conditions for effective and energy‐efficient extraction.

A comparison of the protein yields obtained from different species under different extraction conditions indicated a significantly higher protein yield (*p* < 0.05) for ultrasound‐treated (80 W, 20 kHz, and 20 min) samples compared to the untreated samples and soybean showing the highest yield (Figure 1).

A protein yield increase of 10.92% was observed for ultrasound‐treated *A. cyclops*, 6.37% for *A. microbotrya*, and 7.84% for soybeans. A significant increase in the protein yield during ultrasound‐assisted extraction was also observed by Byanju et al. (2020), with the yield from soy flakes rising from 8.4% to 33.45% at a power density of 2.5 W/cm^2^. However, for soy flour, only a moderate increase in protein yield was observed after ultrasound treatment—from 43% to 50%—which was not significant (*p* > 0.05) (Byanju et al. 2020). Aguilar‐Acosta et al. (2020) also reported that the lupin protein yield treated with the alkaline ultrasonication technique was 14% higher than without ultrasonication treatment. Ultrasound‐assisted extraction increases protein yield by acoustic cavitation, characterized by the formation and collapse of microbubbles, which generate localized high temperatures around 5000 K and pressure around 1000 atm (Malik and Saini 2018). These effects generate intense shear forces, shockwaves, and microjets that rupture cell walls and membranes, facilitating the release of intracellular proteins (Jadhav et al. 2024; Suchintita Das et al. 2023). In addition, ultrasound‐assisted extraction enhances mass transfer between the solid and solvent matrix by increasing solvent penetration and increasing surface area exposure for interaction, thereby reducing diffusion barriers (Durbha and Aravamudan 2012; Jadhav et al. 2024). It may also induce partial unfolding of the protein's tertiary and quaternary structures, which increases extractability (Jadhav et al. 2024). Therefore, the combination of molecular modification and mechanical disruption accounts for increased protein yield (Suchintita Das et al. 2023).

For commercial protein production, maximizing yield is essential. The protein yield is affected by several principal factors, such as the flour's particle size and protein composition (Higa et al. 2022). In this research, the use of particle size <500 µm was intended to increase the rate of protein extraction by increasing the surface area for mass transfer and enhancing solvent penetration. For example, Jahan et al. (2022) demonstrated that reducing the particle size of mustard flour from 600 to 375 µm significantly improved protein yield under ultrasound‐assisted extraction. However, reducing it further to 150 µm decreased the yield.

On a practical level, size reduction is applied for the high extraction rate, but reduced particle size restricts the downstream separation of protein solution from a protein residue. An increased power density can also contribute to the yield; however, the purity of the protein extracted is found to decrease, possibly due to the co‐extraction of other components like oils, sugar, and isoflavones (Vilkhu et al. 2008). To design effective and efficient processes, it is highly desirable to have a fundamental understanding of the effect of microstructures and particle size (Aguilar‐Acosta et al. 2020). Therefore, a particle size <500 µm was chosen in this research as a balance between maintaining downstream processability and protein purity and maximizing the yield.

### 3.2 Functional Properties

#### 3.2.1 Foaming Properties

**FIGURE 2:**
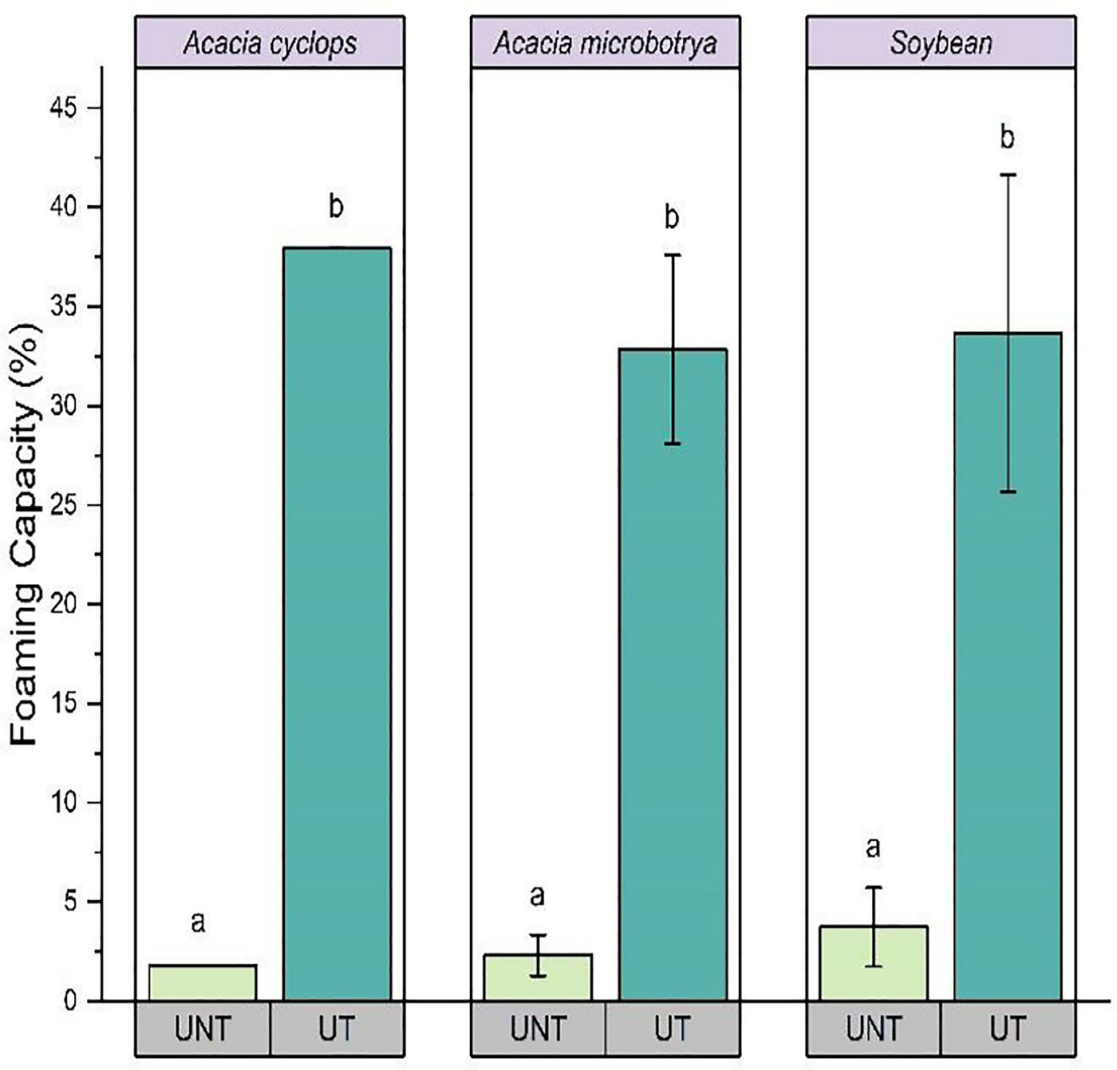
Change in foaming capacity *Acacia cyclops*, *Acacia microbotrya*, and soybean for untreated and ultrasound‐treated conditions. UNT, untreated; UT, ultrasound‐treated (80 W, 20 kHz, 20 min: highest yield condition).

The FC of ultrasound‐treated *Acacia* and soybean proteins was significantly higher compared to untreated proteins (*p* < 0.05), with an increase of 36.15% observed for *A. cyclops*, 30.53% for *A. microbotrya*, and 29.91% for soybean (Figure 2).

The highest FC was obtained for the protein extracted from *A. cyclops* under ultrasound‐assisted conditions. This was possibly due to the enhanced molecular flexibility of *A. cyclops* and surface activity supporting film formation around air bubbles, contributing to the increase FC (Adiamo et al. 2019; Amagliani et al. 2021). Martínez‐Velasco et al. (2018) found a similar increase in the FC of soybean and faba bean protein isolate as a result of ultrasonication and attributed this to the increase in hydrophobicity, volume, and particle size as a result of cavitation.

Likewise, the FS of all the ultrasound‐treated protein samples was significantly higher than that of the untreated protein samples. A higher FS is related to the lower apparent viscosity of treated protein concentrates, which offers decreased resistance to the incorporation of gas bubbles and to the faster rate of adsorption and diffusion of the treated proteins at the air–water interface (Martínez‐Velasco et al. 2018). In this way, minor bubbles were produced that contributed to increased FS. Contrary to the FC results, the ultrasound‐treated soybean showed the highest FS (12.31%), which indicates a lower interfacial tension. Comparable values of FS were observed for proteins extracted from the *Acacia* seeds. However, the overall value of FS in ultrasound‐treated and untreated protein samples (1%–12%) was significantly lower than the results obtained by Kilicli et al. (2023) and Mokni Ghribi et al. (2015) in chickpea concentrate (41.93%–58.06%). Enhanced foaming capacity of treated *Acacia* species supports the potential applications in aerated foods, such as whipped toppings, nougat, and meringue.

#### 3.2.2 Emulsifying Activity Index

**FIGURE 3:**
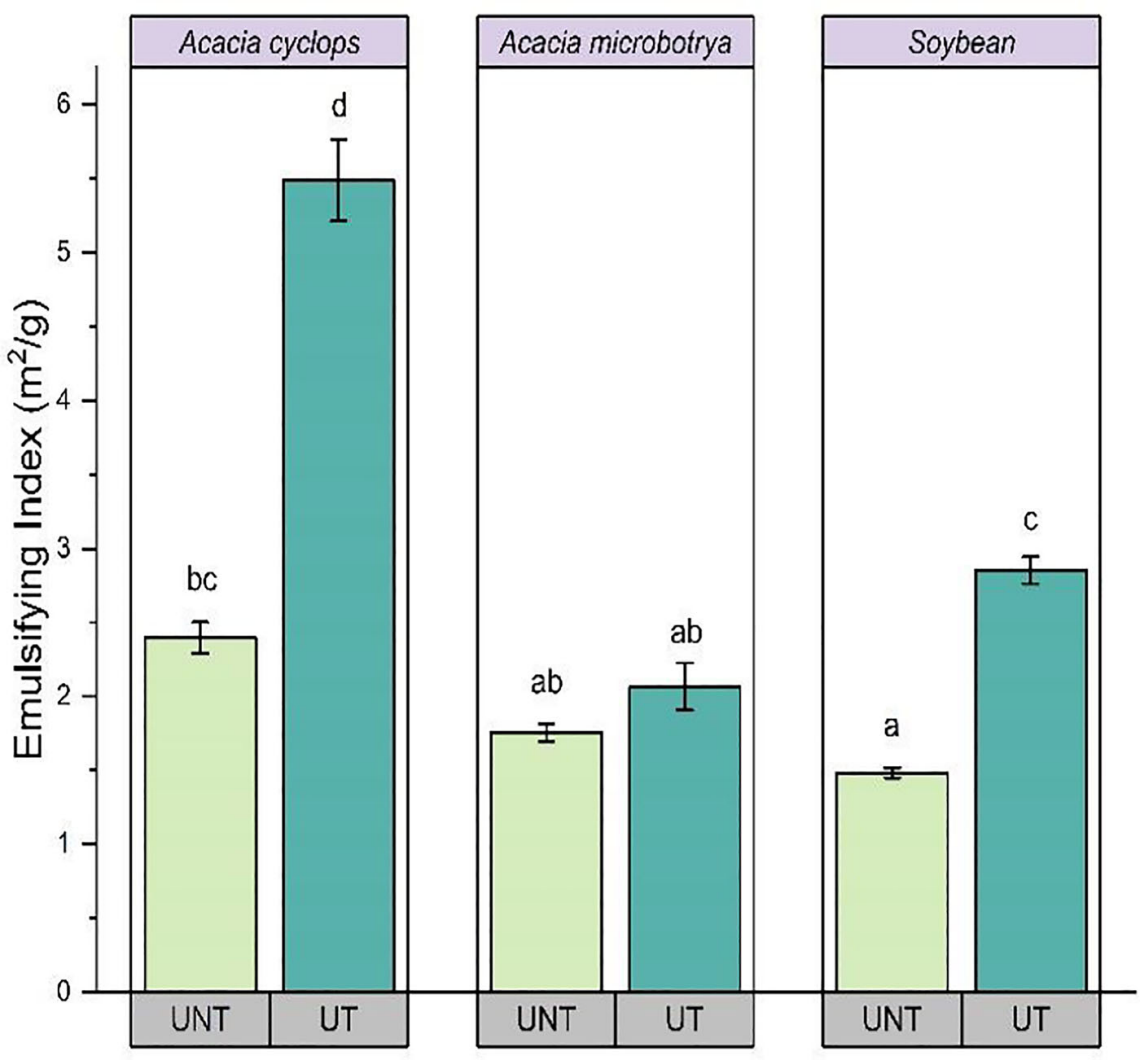
Change in emulsifying index *Acacia cyclops*, *Acacia microbotrya*, and soybean for untreated and ultrasound‐treated conditions. UNT, untreated; UT, ultrasound‐treated (80 W, 20 kHz, 20 min: highest yield condition).

Ultrasound‐assisted extraction increased the EAI for the protein samples studied, as can be seen in Figure 3.

The emulsion activity values obtained for proteins extracted from ultrasound‐treated and untreated *A. cyclops* were 5.48 and 2.39 m^2^/g, respectively, which were significantly higher (*p* < 0.05) than those obtained for *A. microbotrya* and soybean. These differences between ultrasound‐treated and untreated samples in emulsion activities have been attributed to variations in soluble and insoluble protein content (Arzeni et al. 2012). The greater emulsifying activity in sonicated protein samples may also result from partial unfolding and dissociation of globular proteins (tertiary and quaternary structure) due to ultrasound‐induced cavitation (Al‐Ali 2020). Ultrasound treatment is known to alter the myofibrillar protein structures, increasing the surface‐to‐volume ratio that exposes internal hydrophobic residues (Sun et al. 2021). This can elevate the protein's surface activity and improve adsorption at the oil–water interface, with more protein participating in the formation of an interfacial layer and increasing the emulsifying efficiency.

The energy input during ultrasonic treatment would disrupt the non‐covalent bonds (hydrogen and hydrophobic interactions) between proteins, reducing the size of the protein clusters (Alavi et al. 2021). Compared to the emulsifying activity results obtained in this study, pea‐protein isolate demonstrated a higher value of 18.6 m^2^/g under the conditions reported by Cui et al. (2021). This might be due to the variation in structural properties, surface hydrophobicity, solubility, environmental pH, and oil‐to‐water ratio between different legumes (Yan et al. 2024). A protein with improved emulsifying characteristics would have a low MW, a balance of polar and non‐polar amino acids, excellent water solubility, stable conformation, and fully developed surface hydrophobicity (Barac et al. 2015). These findings highlight the potential of legume proteins to function as effective and stable emulsifiers in a wide range of food applications, such as salad dressings and sauces (Mokni Ghribi et al. 2015; Yan et al. 2024).

#### 3.2.3 Water Holding Capacity (WHC) and Oil Holding Capacity (OHC)

**FIGURE 4:**
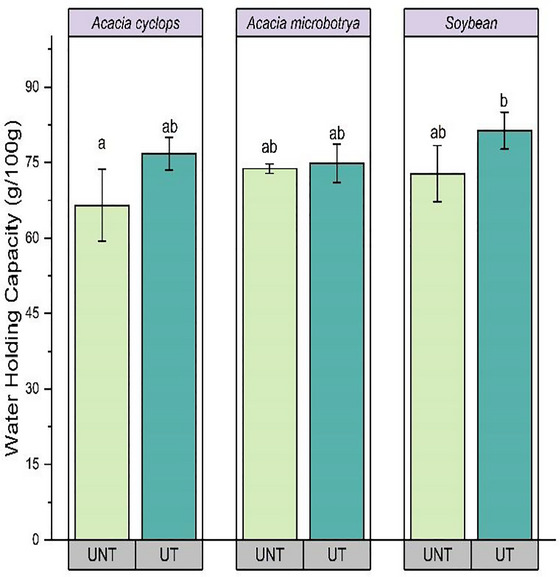
Change in water‐holding capacity *Acacia cyclops*, *Acacia microbotrya*, and soybean for untreated and ultrasound‐treated conditions. UNT, untreated; UT, ultrasound‐treated (80 W, 20 kHz, 20 min: highest yield condition).

The WHC of *A. cyclops* and soybean proteins considerably increased after ultrasound treatment (*p* < 0.05) from 72.80% to 76.78% and 72.80% to 81.34%, respectively (Figure 4).

**FIGURE 5:**
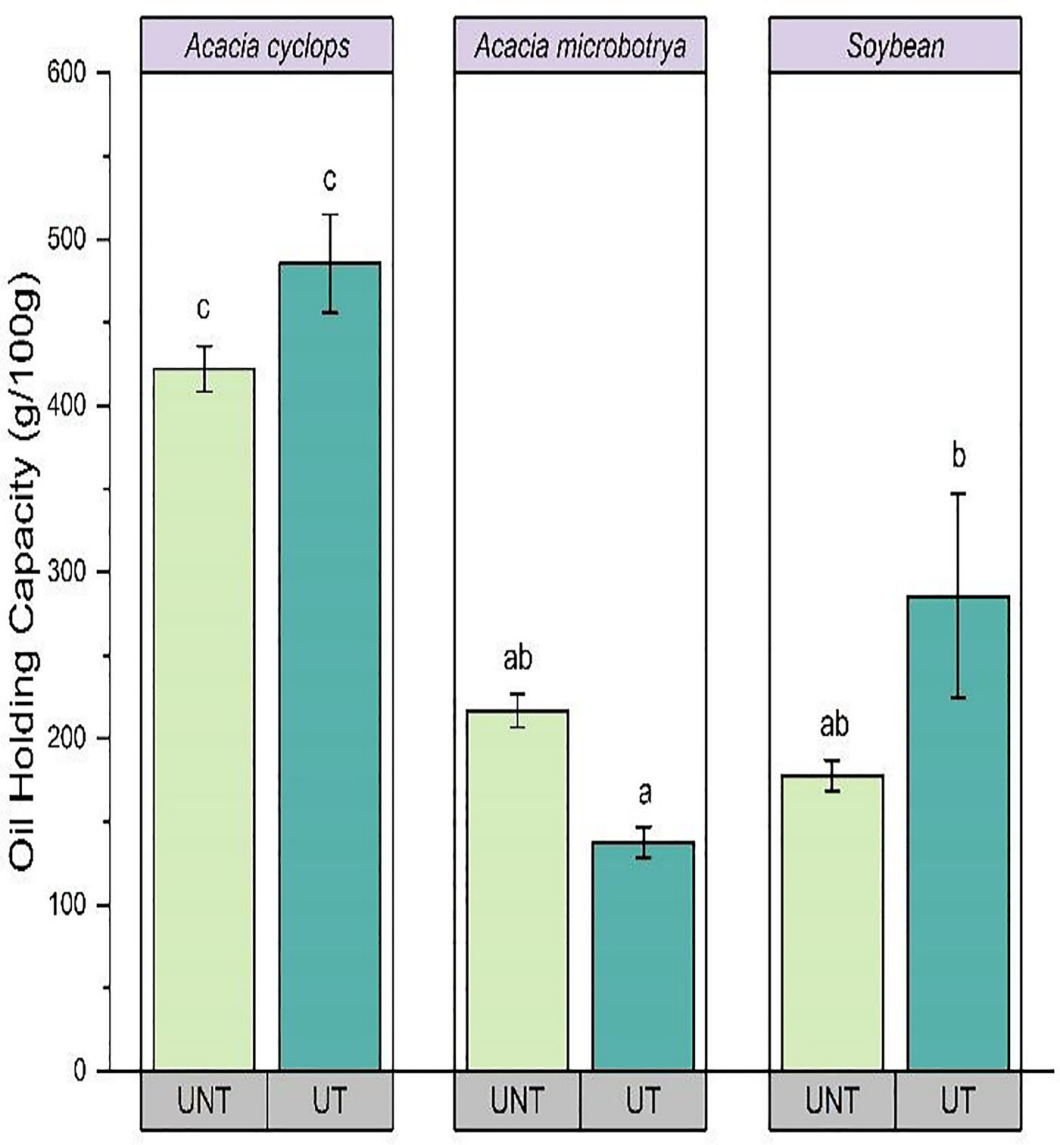
Change in oil‐holding capacity *Acacia cyclops*, *Acacia microbotrya*, and soybean for untreated and ultrasound‐treated conditions. UNT, untreated; UT, ultrasound‐treated (80 W, 20 kHz, 20 min: highest yield condition).

Similar results were reported by Hu et al. (2013), where soy protein concentrate treated with ultrasound at 400 W for 5, 20, and 40 min showed improved WHC. Similarly, the WHC of treated pea‐protein concentrates improved by 40.7% compared to untreated pea‐protein concentrates (Wang et al. 2020). Moreover, the OHC of *A. cyclops* and soybean protein concentrates significantly increased (*p* < 0.05) from 422.12% to 485.40% and 177.71% to 285.72%, respectively (Figure 5).

Ultrasound induces protein unfolding, resulting in the increased exposure of hydrophobic amino acid groups, ultimately enhancing the absorption of oil (Ampofo and Ngadi 2022). Differences in the physical structures of the treated *A. cyclops* protein concentrate, such as high porosity, could allow for a more significant entrapment of oil compared to treated *A. microbotrya* and soybean (Adiamo et al. 2023). The low WHC and OHC of soybean protein concentrate compared to *Acacia* protein concentrates indicate that proteins from *Acacia* could be an appropriate food ingredient in food formulations where hydration properties and oil‐binding properties are necessary, such as in the production of bread and cake (Adiamo et al. 2019).

Protein is the main factor regulating the WHC in food because protein exhibits a dual nature, hydrophobic and hydrophilic (Jitngarmkusol et al. 2008). The WHC in the different protein concentrates ranged between 71.80% and 81.34%. A significantly higher WHC in ultrasound‐treated soybean protein concentrate than *Acacia* proteins (*p* < 0.05) could be due to minimal losses of soluble protein during protein extraction and the availability of higher polar amino acids in soybean proteins (Mokni Ghribi et al. 2015). The lower WHC in *Acacia* protein concentrates could be attributed to the lower content of polar proteins, compositional difference, and protein concentration (Aguilar‐Acosta et al. 2020).

#### 3.2.4 Solubility

**FIGURE 6:**
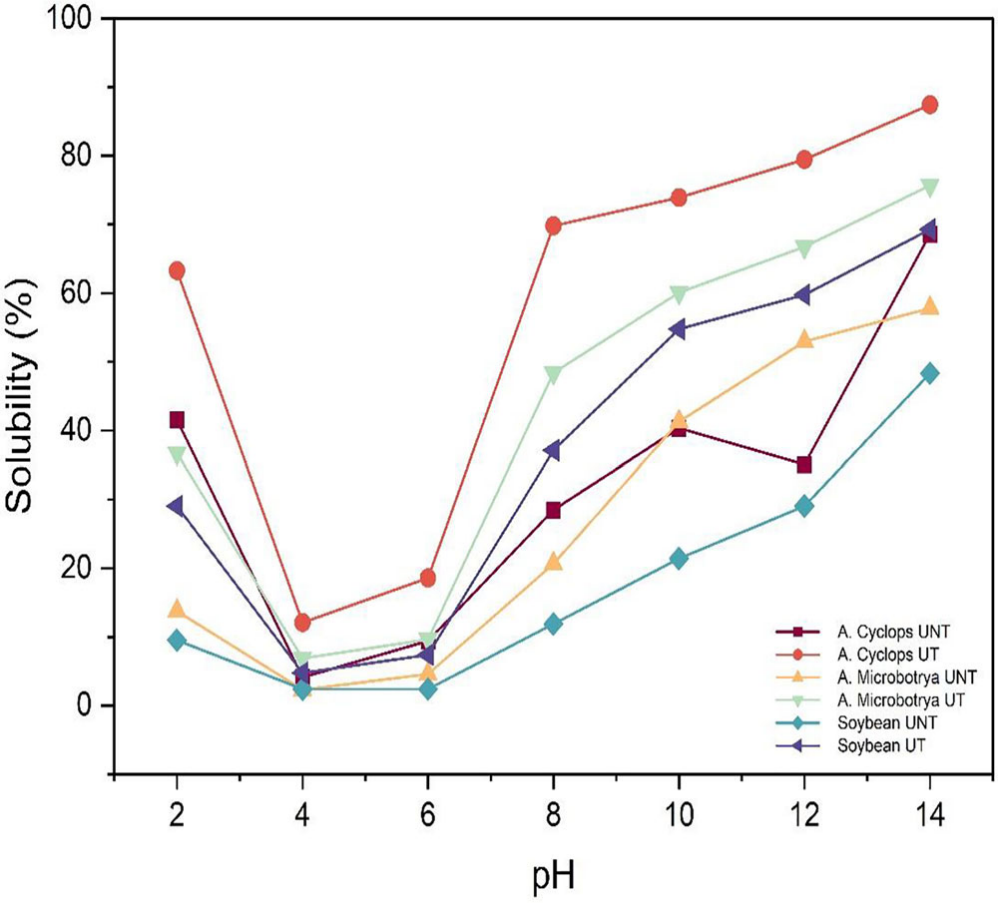
Change in solubility of the protein isolates extracted from *Acacia cyclops*, *Acacia microbotrya*, and soybean for untreated and ultrasound‐treated conditions. UNT, untreated; UT, ultrasound‐treated (80 W, 20 kHz, 20 min: highest yield condition).

The solubility of both ultrasound‐treated and untreated proteins decreased with an increase in pH until the minimum solubility was obtained at pH 4 (Figure 6).

The solubility values obtained at pH 4 were 12.08% and 4.11% for treated and untreated *A. cyclops* protein concentrates, 6.88% and 2.29% in treated and untreated *A. microbotrya* protein concentrates, and 4.76% and 2.38% in treated and untreated soybean protein concentrates, respectively. The solubility increased with the increase in pH after 4, with the highest solubilities obtained at pH 14, ranging from 48.33% to 87.44%. The highest value was for ultrasound‐treated *A. cyclops* protein concentrate. The solubility of ultrasonic‐treated protein concentrates increased significantly (*p* < 0.05) compared to untreated protein concentrates. For instance, 28.5% to 69.82% for *A. cyclops*, 20.64% to 48.39% for *A. microbotrya*, and 11.9% to 37.14% for soybean at pH 8. Proteins are present in the form of aggregates in a natural state; cavitation by ultrasonication may disturb the hydrophobic interactions and hydrogen bonding, thereby reducing the MW of protein aggregates. This reduction in MWs results in a decrease in particle size and the exposure of hydrophilic amino acid groups, consequently amplifying the protein's surface area in a solvent under specific pH conditions, leading to heightened solubility (Ampofo and Ngadi 2022; Zhang et al. 2017). Greater solubility is an indication of low denaturation and native proteins (Yousefi and Abbasi 2022). Similar results were observed by Arzeni et al. (2012), where ultrasound‐treated soy protein concentrate with a 20 kHz probe showed a significant increase in protein solubility compared to untreated soy protein concentrate.

Solubility is a reliable index of protein's techno‐functionality and the most practical measurement of protein aggregation and denaturation. Protein solubility is important in food systems because it affects other techno‐functional properties. Therefore, any enhancement of commercial protein solubility has the potential to enhance other functional properties. Most plant proteins have greater solubility above pH 7. The solubility is moderately decreased in acidic pH (3.5–5) (Adiamo et al. 2023). Similar results were reported in protein extracts of other legumes, such as soybean, cowpea, faba beans, and lentil, where protein solubility increased when the pH changed from acidic to alkaline (Adiamo et al. 2019).

#### 3.2.5 In Vitro Protein Digestibility

Understanding the IVPD of protein concentrates is important for evaluating their bioavailability and nutritional quality, which are essential for commercial purposes, especially for plant proteins (Shaghaghian et al. 2022). In this study, ultrasound treatment significantly increased the IVPD of *Acacia* and soybean protein concentrates by over 10% (*p* < 0.05), with values ranging from 82.20% to 96.72%. This result aligns with the previous research demonstrating an increase in IVPD with ultrasound treatment when legume proteins, such as soybean and chickpea, were treated with ultrasound (Khatkar et al. 2020; Wang et al. 2020). Khatkar et al. (2020) showed IVPD of sonicated soybean proteins increased significantly (*p* < 0.05) from 86.17% to 96.88%. This enhancement is a result of ultrasound‐induced unfolding of protein structures. Ultrasound can cause conformational changes in proteins and disrupt various intramolecular interactions. These changes expose hydrophobic amino acid residues and cleavage sites, making them accessible for enzymatic action. This structural modification thereby increases the IVPD of the treated protein concentrates (Villarino et al. 2015). Incorporating these findings into commercial applications can facilitate the development of plant‐based protein products with increased nutritional profiles.

#### 3.2.6 FTIR Analysis

**FIGURE 7:**
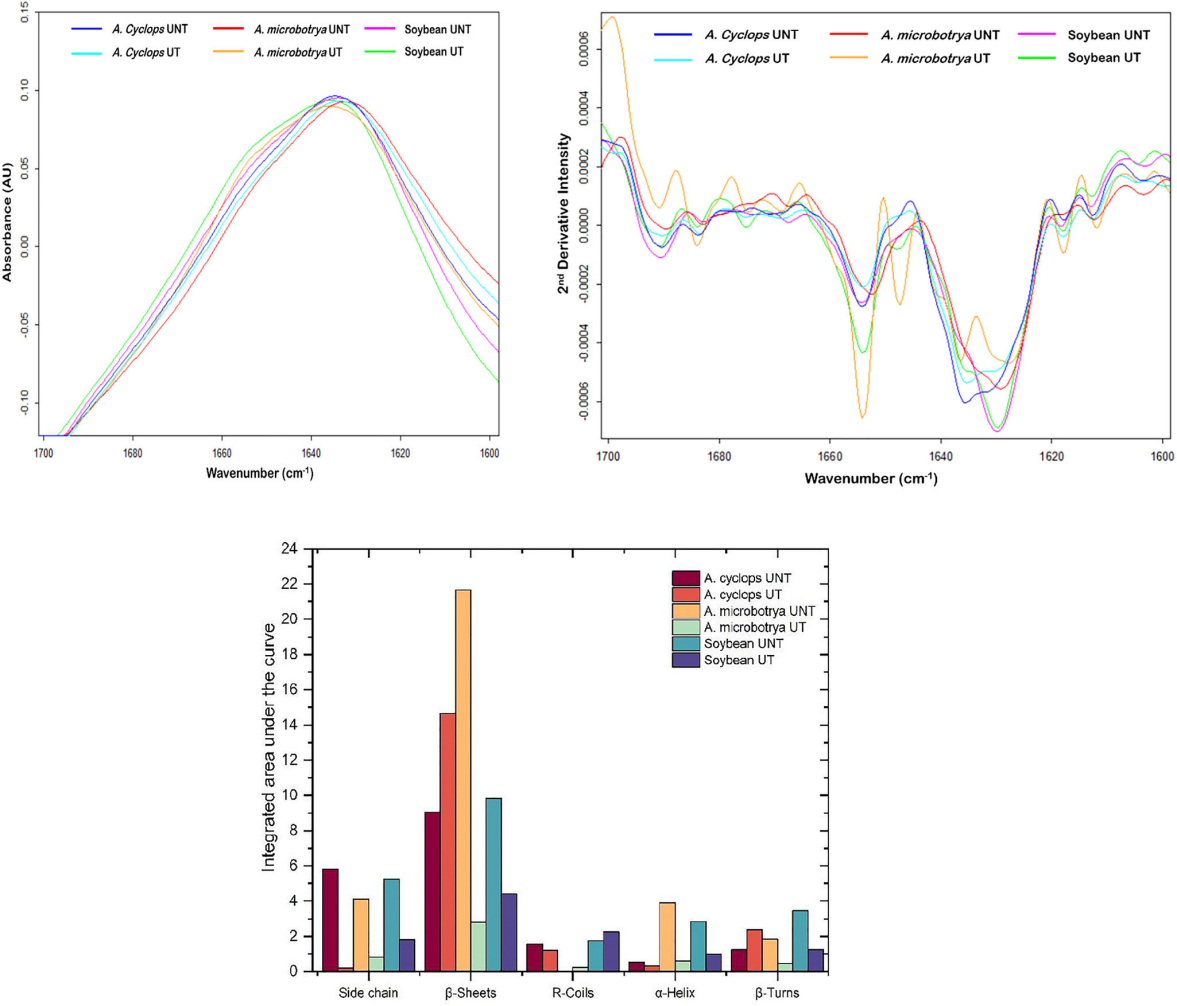
(a) ATR‐FTIR baseline corrected spectra of proteins showing the Amide I region. (b) Second derivative spectra of the Amide I region. (c) Contribution of different conformational peaks.

Figure 7a shows the complete Amide I region, and minor differences in the peak intensities between ultrasound‐treated and untreated samples were observed. However, the single broad peak of the Amide I (1600–1700 cm^−1^) band is the result of multiple overlapping absorbance bands, each representing an underlying secondary structure (Shah et al. 2021).

The positions of the underlying bands were identified through spectral deconvolution approaches, such as the calculation of second derivatives (Figure 7b). These locations were identified as 1690, 1683, 1675, 1667, 1654, 1647, 1629, 161, and 1612 cm^−1^, of which a significant difference in the intensities was observed at 1654 cm^−1^. Curve fitting was performed using a linear least squares algorithm by including two additional bands to those identified from second derivatives to account for absorbance contribution from the neighboring ester carbonyl and Amide II bands. The assignment of each Gaussian component to a specific secondary structure was conducted according to Carbonaro et al. (2012) and Kłosok et al. (2021).

Figure 7c represents the contribution of different conformational peaks in the Amide I region for the samples studied, and a significant alteration in the contribution of various protein secondary structures was observed following ultrasound treatment. The native protein of legumes has a secondary structure dominated by β‐sheets and β‐turn structures (Aguilar‐Acosta et al. 2020). Agboola and Aluko (2009), upon analysis of the ultraviolet‐circular dichroism spectra of *Acacia victoriae* species, revealed that the protein consists mainly of equal values (39%) of β‐sheets and random structures, followed by β‐turns (19%) and relatively low amount of α‐helix (3.6%). In the present study, a similar result was observed in the case of β‐sheets, which showed maximum proportion, with the highest content found in protein extracted from untreated *A. microbotrya*. However, upon sonication, a significant decrease in β‐sheets was observed. A similar observation was made for soybean; however, for *A*. *cyclops*, ultrasound treatment resulted in an increase in β‐sheets. A comparable observation was made for β‐turns, whereas ultrasound treatment caused a reduction in sidechains and α‐helices. Kang et al. (2022) observed a significant reduction in the level of α‐helix and random coils in chickpea protein concentrate following ultrasound treatment, whereas the β‐structure was found to increase. They explained this observation as a result of the cavitation phenomenon that destroys the hydrogen bonding between the carbonyl and amino groups on the polypeptide chain, thereby stabilizing the α‐helix structure and increasing β‐structure. They also suggested the possibility of transformation of random coiled structures into β‐sheet structures with long‐term sonication as a result of protein cross‐linking. Similar results were obtained by Rafique et al. (2025) on oat protein isolates and explained it as a result of expansion of secondary structure of protein due to its microstructure breakdown. Interestingly, a decrease in α‐helix units and an increase of β‐sheets are also associated with more interior hydrophobic interactions, which in turn facilitates the molecular diffusion of these functional groups (α‐helix and β‐sheets) on the surface of smaller soluble particles in order to better adsorb oil droplets at the oil–water interface (Gharibzahedi and Smith 2020). This can explain the significant increase in the emulsifying index of protein extract from ultrasound‐treated *A. cyclops* as compared to other samples (Figure 3). Overall, ultrasound was found to result in significant changes in the secondary structures of *Acacia* species studied, which could influence the functional properties of the proteins.

#### 3.2.7 Electrophoresis

**FIGURE 8:**
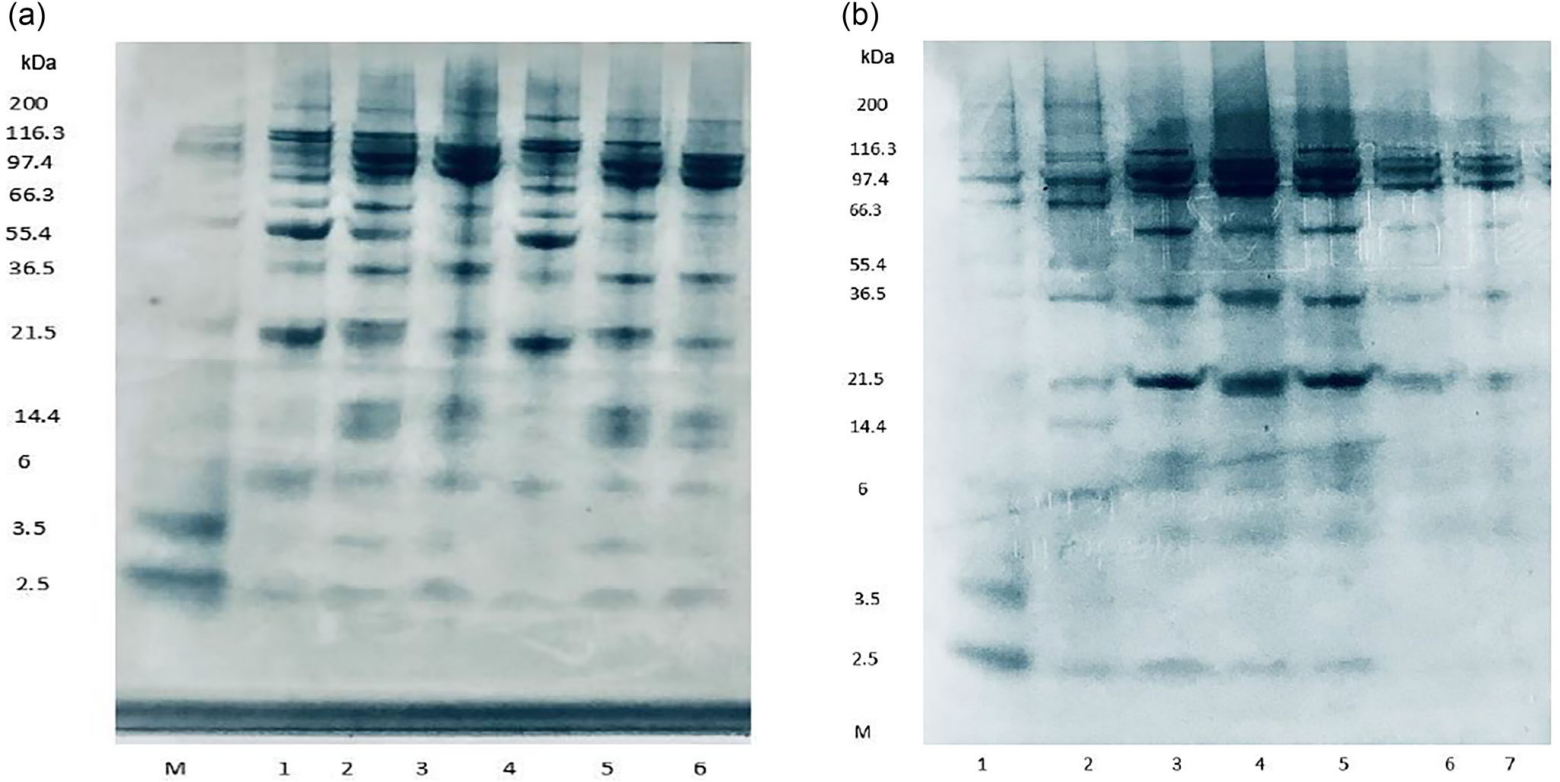
(a) SDS–PAGE of the proteins under reducing condition (M: molecular weight marker, 1: SCR, 2: STR, 3: ACCR, 4: ACTR, 5: AMCR, 6: AMTR). (b) SDS–PAGE of the proteins under non‐reducing condition (M: molecular weight marker, 1: SCNR, 2: STNR, 3: ACCNR, 4: ACTNR, 5: AMCNR, 6: AMTNR). ACCNR‐*Acacia cyclops* control non‐reducing; ACCR‐*Acacia cyclops* control reducing; ACTNR‐*Acacia cyclops* treated non‐reducing; ACTR‐*Acacia cyclops* treated reducing; AMCNR‐*Acacia microbotrya* non‐reducing; AMCR‐*Acacia microbotrya* control reducing; AMTNR‐*Acacia microbotrya* treated non‐reducing; AMTR‐*Acacia microbotrya* treated reducing; SCNR‐soybean control non‐reducing; SCR‐soybean control reducing; STNR‐soybean treated non‐reducing; STR‐soybean treated reducing.

Electrophoretic analysis serves as an essential technique in this study to elucidate the structural modification induced by ultrasound treatment on the *Acacia* and soybean protein concentrates. Understanding the structural modifications is critical, as they influence the bioavailability and functional properties of plant proteins, which are essential factors in their commercial application (Badjona et al. 2024). Electrophoretic separation of ultrasound‐treated and untreated protein concentrates of two varieties of *Acacia* and soybean under both reducing and nonreducing conditions is shown in Figure 8. Under reducing conditions, the predominant bands were detected at approx. 90, 66, and 23 kDa, which corresponds to the fraction of convicilin, minor legumin subunit, and 11S legumin β chain, respectively (Gulzar et al. 2024; Le Signor et al. 2005).

Under the nonreducing condition, band separation is predominant at 97 kDa, possibly attributed to convicilin. A minor band emerged at 35 kDa, corresponding to the acidic legumin subunit (Żmudziński et al. 2021). After ultrasound treatment (Figure 8b, lanes 2, 4, and 6), under non‐reducing conditions, there is a reduction in the intensity of the legumin unit band (lanes 1, 3, and 5) compared to untreated protein concentrates at approx. 35 kDa, indicating the partial fragmentation of legumin subunits by ultrasound (Gulzar et al. 2024). However, band intensity remained unchanged at 97 kDa across all protein concentrates, and high MW aggregates appeared as smears at the head of the SDS–PAGE gel. This is because the dissociated polypeptides are aggregated via a disulfide bond (Alavi et al. 2021). A similar appearance was reported by Gulzar et al. (2024) in ultrasound‐treated faba bean protein concentrate. Under reducing conditions for both ultrasound‐treated *Acacia* protein concentrates (Figure 8a, lanes 4 and 6), a progressive intensity reduction in the polypeptide bands around 36 kDa was observed. This indicates that disulfide bonds play a significant role in the formation of aggregates. Overall, it was observed that the ultrasound treatment caused prominent alterations in all protein concentrates that have an effect on protein functionality (Gulzar et al. 2024).

## 4 Conclusion

Ultrasound significantly improved the extraction yield and functional properties of *Acacia* and soybean protein concentrates. The increased yield and techno‐functionality of ultrasound‐treated protein concentrates observed in this study can be attributed to molecular and structural modifications induced by ultrasound treatment. Under optimal conditions (80 W, 20 kHz, 20 min), protein yields increased by 10.92%, 6.37%, and 7.84% for *A. cyclops*, *A. microbotrya*, and soybean, respectively, compared to their untreated counterparts. The application of ultrasound led to a considerable improvement in key functional properties. Treated *A. cyclops* protein concentrates exhibited the highest foaming capacity (37.93%), EAI (5.48 m^2^/g), IVPD (96.72%), OHC (485%) (*p* < 0.05), outperforming both soybean and *A. microbotrya* protein concentrates. Likewise, treated soybean protein concentrates showed significant increases (*p* < 0.05) in FS, WHC, and protein yield compared to untreated protein concentrates. Despite the improvements observed in ultrasound‐treated protein concentrates, some untreated samples retained superior functionality. The WHC of untreated *A. microbotrya* protein concentrate was the highest of all the protein concentrates (73.80%), whereas untreated *A. cyclops* exhibited higher EAI (2.39 m^2^/g) and OHC (422.12%) than untreated *A. microbotrya* and soybean. Moreover, untreated soybean protein concentrates had the highest FC, FS, and protein yield, surpassing untreated *A. cyclops* by 8.52% and *A. microbotrya* by 13.93%.

## Author Contributions

**Laxmi Ghimire**: writing–original draft, writing–review and editing, formal analysis, methodology, data curation, visualization. **Nedumpillil Unnikrishnan Sruthi**: software, writing–review and editing, formal analysis, data curation, visualization. **Simon Warwick**: writing–review and editing. **Ranil Coorey**: resources, writing ‐ review and editing. **Rewati Raman Bhattarai**: conceptualization, supervision, funding acquisition, project administration, writing ‐ review and editing.

## Conflicts of Interest

The authors declare no conflicts of interest.
